# The Lateral Epidermis Actively Counteracts Pulling by the Amnioserosa During Dorsal Closure

**DOI:** 10.3389/fcell.2022.865397

**Published:** 2022-05-16

**Authors:** Zhiyi Lv, Na Zhang, Xiaozhu Zhang, Jörg Großhans, Deqing Kong

**Affiliations:** ^1^ Institute of Evolution and Marine Biodiversity, Ocean University of China, Qingdao, China; ^2^ Department of Biology, Philipps University, Marburg, Germany; ^3^ MOE Key Laboratory of Advanced Micro-Structured Materials and School of Physics Science and Engineering, Tongji University, Shanghai, China; ^4^ Frontiers Science Center for Intelligent Autonomous Systems, Tongji University, Shanghai, China; ^5^ Institute for Theoretical Physics and Center for Advancing Electronics Dresden (cfaed), Cluster of Excellence Physics of Life, TU Dresden, Dresden, Germany

**Keywords:** morphogenesis, dorsal closure, tissue mechanics, cell shape change, microtubules, optochemistry

## Abstract

Dorsal closure is a prominent morphogenetic process during *Drosophila* embryogenesis, which involves two epithelial tissues, that is, the squamous amnioserosa and the columnar lateral epidermis. Non-muscle myosin II-driven constriction in the amnioserosa leads to a decrease in the apical surface area and pulls on the adjacent lateral epidermis, which subsequently moves dorsally. The pull by the amnioserosa becomes obvious in an elongation of the epidermal cells, especially of those in the first row. The contribution of the epidermal cell elongation has remained unclear to dorsal closure. Cell elongation may be a mere passive consequence or an active response to the pulling by the amnioserosa. Here, we found that the lateral epidermis actively responds. We analyzed tensions within tissues and cell junctions by laser ablation before and during dorsal closure, the elliptical and dorsal closure stages, respectively. Furthermore, we genetically and optochemically induced chronic and acute cell contraction, respectively. In this way, we found that tension in the epidermis increased during dorsal closure. A correspondingly increased tension was not observed at individual junctions, however. Junctional tension even decreased during dorsal closure in the epidermis. We strikingly observed a strong increase of the microtubule amount in the epidermis, while non-muscle myosin II increased in both tissues. Our data suggest that the epidermis actively antagonizes the pull from the amnioserosa during dorsal closure and the increased microtubules might help the epidermis bear part of the mechanical force.

## Introduction

Tissue morphogenesis requires cellular activities such as cell shape changes (Leptin, 1994; Lye and Sanson, 2011; Kong et al., 2017; Lv et al., 2019). Cells change their shape by autonomously generating intracellular mechanical forces (Roh-Johnson et al., 2012; Blanchard et al., 2018) and (or) responding to external mechanical forces from neighboring cells within the tissue; or by forces from the neighboring tissues (Bhide et al., 2021). During embryogenesis, mechanical forces triggered cellular activities coordinatively in the neighboring compartments are required for an efficient morphogenetic process (Collinet et al., 2015; Lye et al., 2015; Kong et al., 2017; Sharrock and Sanson, 2020; Villedieu et al., 2020; Zhao et al., 2021). How cell and tissue mechanics are regulated and integrated to affect changes in cell shape is essential to describe the mechanisms of morphogenesis.

**FIGURE 1:**
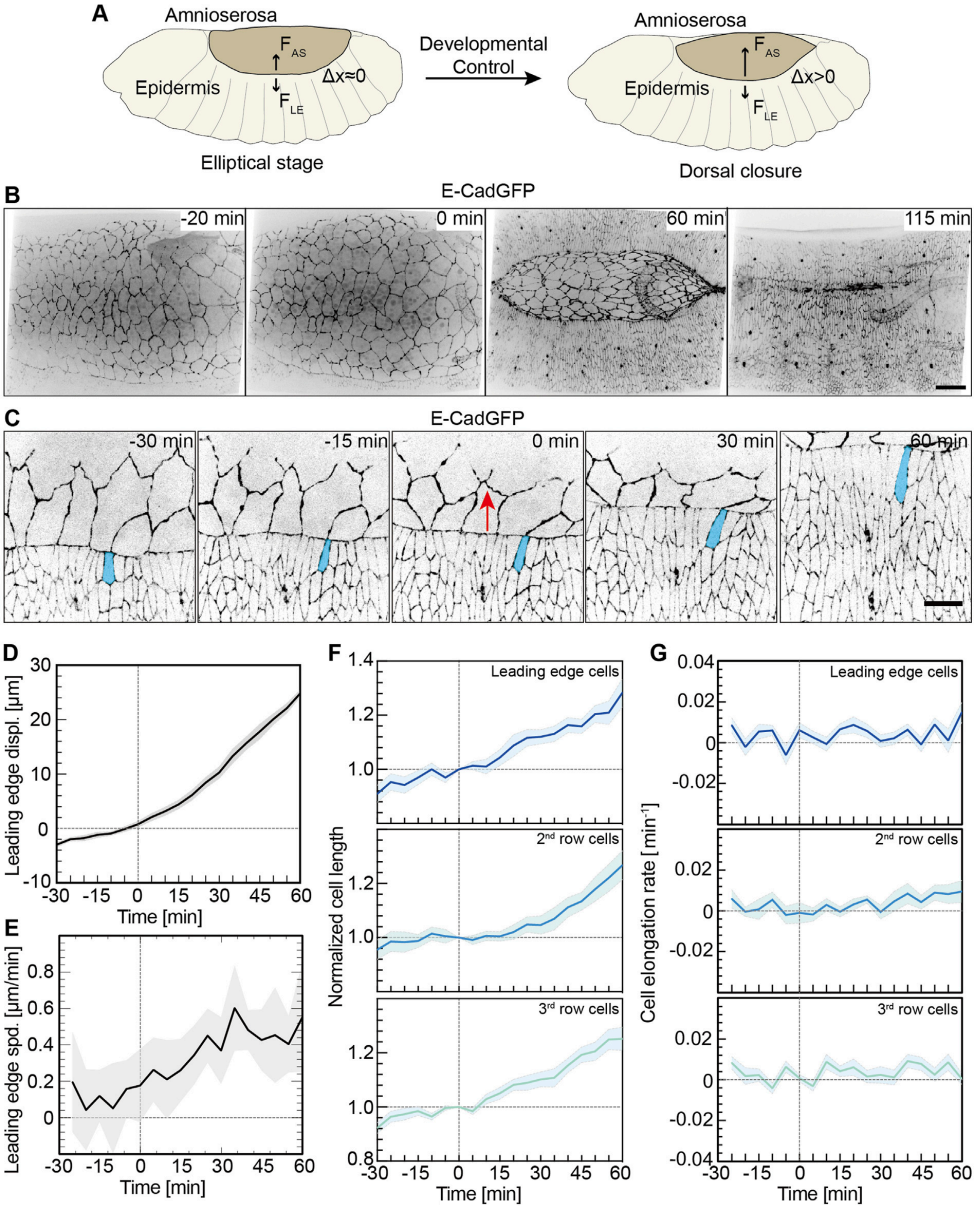
Lateral epidermal cells elongate in a pulsatile manner. **(A)** Schematic representation of a *Drosophila* embryo in the ellipsoidal stage and undergoing dorsal closure. F_AS_, the AS contractile forces; F_LE_, forces from the lateral epidermis; and △x, the lateral epidermis movements dorsally. **(B)** Images from a time-lapse recording in an embryo expressing E-CadGFP undergoing dorsal closure (0 min, onset of dorsal closure with the noticeable displacement of leading-edge dorsally). Scale bar is 25 μm. **(C)** Images from a time-lapse recording of an embryo expressing E-CadGFP show that the surrounding epidermal cells elongate along the dorsal–ventral axis during dorsal closure; 0 min, onset of dorsal closure with the noticeable displacement of the leading edge dorsally. A leading-edge epidermal cell is highlighted in blue. The red arrows indicate the movements of the leading edge during dorsal closure. Scale bar is 10 μm. **(D,E)** Quantification of the leading-edge displacements dorsally over 90 min. The time is consistent with panel **(C)**. The leading-edge position at 0 min was defined as 0 for measurements. Mean (bold line) with SD (ribbon band) of the mean. *N* = 9 from three embryos, three independent measurements from each embryo. The instantaneous speed of leading-edge displacements is calculated in panel **(E)** from the data in panel **(D)**. **(F,G)** Cell length change of the dorsal-most epidermal (DME) cells along with lateral orientation over 90 min. The cell length was measured along with the lateral direction and normalized to 1 at 0 min. Mean (bold line) with SEM of the mean (ribbon band). *N* = 15 cells in each row from three embryos, five independent cells in each row in each embryo. The instantaneous speed of the lateral epidermal cell elongation is calculated in panel **(G)**.

Dorsal closure (DC) is a prominent morphogenetic process during *Drosophila* embryogenesis. DC includes epithelial sheet movements and fusion (Hayes and Solon, 2017; Kiehart et al., 2017; Manning et al., 2019). For DC, two distinct epithelial tissues interact, that is, the squamous amnioserosa (AS) and the columnar lateral epidermis (Figure 1A). The AS resides on the dorsal side of the embryo and bridges the left and right sheets of the lateral epidermis. AS surface area remains relatively constant during the elliptical stage, the stage from germband retraction to the onset of DC (about 1–1.5 h) (Kiehart et al., 2017) (Figure 1A). The onset of DC begins with a movement of the lateral epidermis toward the dorsal midline (Kiehart et al., 2017). Within about 2 hours, the lateral epidermal sheets on the left and right sides of the embryo move toward and fuse at the dorsal midline, while the AS retreats and finally disappears. A supra-cellular actomyosin cable forms at the interface between AS and the lateral epidermis at the onset of DC within each epidermal cell, and the interface resolves itself into a smooth arc (Young et al., 1993; Kiehart et al., 2000; Jacinto et al., 2002a; Kaltschmidt et al., 2002).

The contributions from AS cells and actin cable for the closure process were described in great detail (Hayes and Solon, 2017; Hutson et al., 2003; Kiehart et al., 2017; Kong and Großhans, 2020; Manning et al., 2019). The mechanical forces provided from both tissues and their interface are required to achieve the closure process (Figure 1A) (Gorfinkiel, 2013; Hayes and Solon, 2017). The AS cells display pulsatile isotropic contractions, which lead to cross-sectional area oscillations, generating pulsed forces by a ratchet-like mechanism and driving the directed movement for closure combined with actin cable tension (Solon et al., 2009). Recently, it has been revealed that the AS cell contraction autonomously drives DC, while the actin cable is dispensable in the direction of DC dynamics but neutralizes mechanical stress (Ducuing and Vincent, 2016; Pasakarnis et al., 2016). Yet, the closure process also involves the movement of the lateral epidermis (Figure 1B). The lateral epidermal cells elongate along the dorsal–ventral direction during DC, especially the leading-edge cells (Kiehart et al., 2000; Jacinto et al., 2002b). The contribution of the lateral epidermal cell shape change for DC has remained unclear. Cell shape change may be a passive consequence of DC; for example, AS contraction generates pull forces stretching the lateral epidermis. Alternatively, the lateral epidermal cells may change their shapes and actively participate in the cell sheet forces; that is, the epidermal cells may generate a counterforce to pulling by the AS and thus actively contribute to the speed and timing of DC.

In this study, we investigated the cellular dynamics of the lateral epidermis during DC. We induced extracontractility in the epidermis chronically by Rho1 pathway activation and acutely by CaLM. We also measured non-muscle myosin II levels and the tension of cells and tissue prior to and during DC. In contrast to non-muscle myosin II, which increases in the epidermis as much as in the AS, we find a specific and striking increase of microtubules in the epidermis during DC. Our results suggest that the lateral epidermis may actively counteract pulling by the AS during DC.

## Results

### Pulsed Elongation of Lateral Epidermal Cells Along with Lateral Orientation During Dorsal Closure

As previously reported, cells of the lateral epidermis elongate toward the AS during DC, especially at the leading edge (Figure 1B and Supplementary Video S1) (Kiehart et al., 2000; Jacinto et al., 2002b). The kinetics of cell elongation has not been thoroughly quantified. Based on time-lapse recordings of embryos expressing E-cadherin-GFP (E-CadGFP), we measured the displacement and elongation of epidermal cells (three rows of cells including leading-edge cells and dorsal-most epidermal cells) (Figure 1C) over about 90 min prior and during DC (Figure 1C and Supplementary Video S2). The measurements were aligned to the onset of DC (t = 0), as defined by the noticeable displacement of the leading edge dorsally. The leading edge moved dorsally slightly with approximately 2 μm in 30 min before the onset of DC and more than 25 μm in 60 min during DC (Figure 1D). We obtained an approximately linear displacement over time (Figure 1D). We calculated the rate of displacements and obtained an increasing rate, especially in the first 30 min of DC (Figure 1E). The length of epidermal cells increased almost linearly (Figure 1F). On average, the cell length extended by 1.3-fold in 60 min during DC (Figure 1F). Plotting the elongation speed revealed pulsatile kinetics (Figures 1F,G).

### Induced Contraction of Amnioserosa Cells Triggers Leading-Edge Displacement

Previous studies have shown that the contraction of AS cells is required for DC (Solon et al., 2009; Pasakarnis et al., 2016). Here, we asked whether the increased contractility of a single cell would suffice for increased pulling by the AS. We also aimed to estimate the reach of such a pulling force, given the viscoelastic nature of the tissue. We previously described an optochemical approach that controls epithelial cell contractility via Ca^2+^-mediated light activation of non-muscle myosin II (CaLM) on the scale of seconds and at single-cell resolution during tissue morphogenesis (Kong et al., 2019).

**FIGURE 2:**
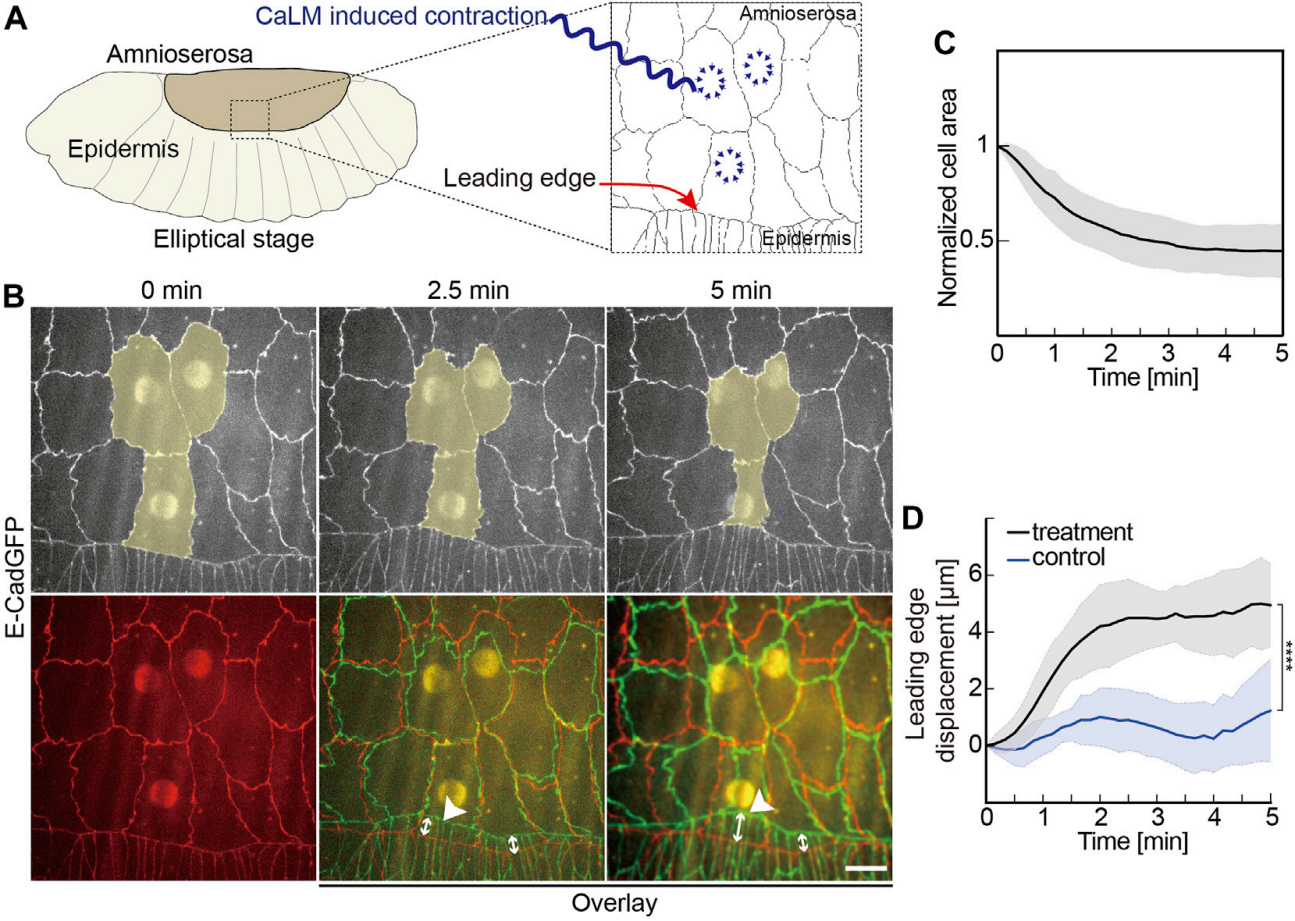
Induced contractility in the AS promotes dorsal closure. **(A)** Experimental scheme of optochemically (CaLM, Ca^2+^ uncaging by UV illumination) induced contraction of AS cells at the elliptical stage in *Drosophila* embryos. The target cells of UV illumination are blue, where the cell contractility is triggered optochemically. **(B)** Images from a time-lapse recording of an embryo expressing E-CadGFP after optochemically induced contraction in target AS cells. Target cells are highlighted in yellow. Image at 0 min is the first frame of a time-lapse recording after UV-laser illumination. The bottom panel shows images’ overlay at 2.5 and 5 min (green) with the image at 0 min (red). White arrow lines indicate the leading-edge displacements. **(C)** Time courses of the cross-sectional area of the target cells over 5 min after UV-laser illumination. Cell areas were normalized to their initial size at 0 min. Five independent experiments from five embryos, *N* = 15 target cells from five embryos, and three target cells from each embryo. **(D)** Quantification of the leading-edge displacement dorsally over 5 min after UV-laser illumination. The leading edge far away from the optochemically induced contracting AS cells in the same embryo was used as control. Five independent experiments from five embryos, two-way ANOVA, Sidak’s multiple comparison test, ****p < 0.0001. Curves represent the mean (bold line) with SD (ribbon band) in panels **(C,D)**.

We applied CaLM and targeted AS cells at elliptical stage embryos expressing E-CadGFP and inspected the movement of the leading edge (Figure 2 and Supplementary Figure S1). Applying CaLM to a single AS cell induced the rapid cell contraction to half of the cross-section area but no obvious displacement of the leading edge (Supplementary Figure S1). In contrast, when we simultaneously applied CaLM in three neighboring AS cells (Figures 2B,C, Supplementary Video S3), we measured a displacement of about 5 μm dorsally in 5 min, significantly higher than in control (Figures 2B,D). Control displacement was measured from the same embryos but away from the contracting AS cells.

**FIGURE 3:**
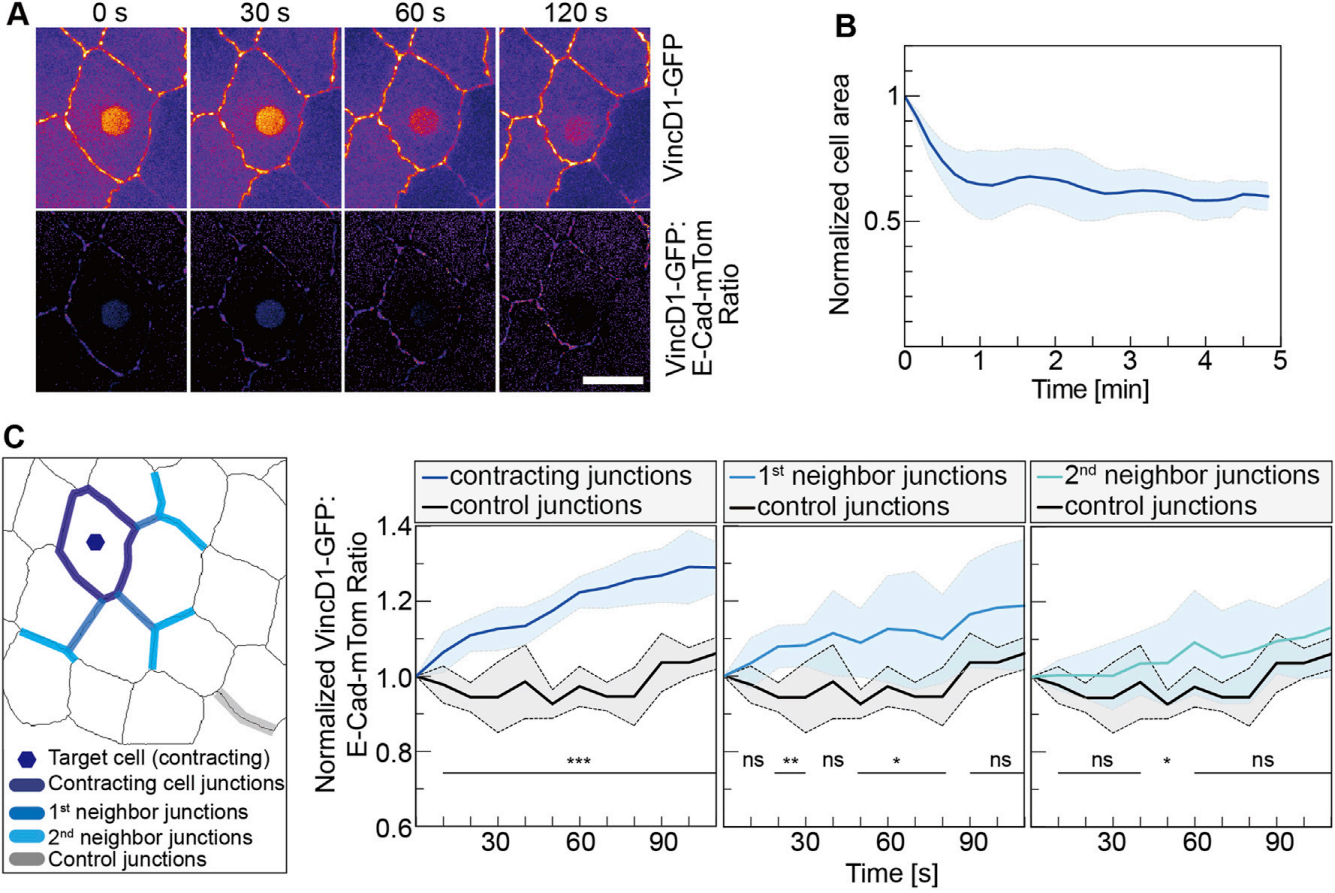
Mechanical pull by a contracting cell is spread to neighbor junctions. **(A)** VincD1-GFP channel and ratiometric images from time-lapse recordings of embryos (stage 14, AS) expressing VincD1GFP and E-CadmTom after UV-laser illumination for 500 ms. **(B)** Time courses of normalized cross-sectional area and its derivative of the target cells (*N* = 6). **(C)** Time course of the ratiometric VincD1-GFP signal averaged of junctions, as indicated in the scheme. Junctions are grouped into three categories: junctions of the target cell (*N* = 30), next to the target cell (primary, *N* = 18), and next-next to the target cell (secondary, *N* = 18). Controls were the junctions (*N* = 5) most distant to the target cell from movies of five embryos. Curves represent mean (bold line) with SD (ribbon band). Unpaired two-sided *t*-test estimates the *p* values, ns, not significant, *p < 0.05, **p < 0.01, and ***p < 0.001. Scale bar, 10 μm.

We employed a Vinculin biosensor to assay the force distribution next to the contracting cells (Kong et al., 2019) and obtained an estimation for the reach of the pulling force by the contracting cell. This biosensor is based on the force-dependent conformational change of alpha-Catenin (alpha-Cat), a component of adherence junctions and binding partner of E-Cad (Choi et al., 2012; Rangarajan and Izard, 2012; Yao et al., 2014; Duong et al., 2021). In the closed state, the central mechanosensitive modulatory (M) domain of alpha-Cat is inaccessible to the D1 domain of Vinculin. In contrast, the stretching of alpha-Cat induces a conformational change, which exposes the M domain and allows binding of the D1 domain. alpha-Cat bridges the cadherin complex with the actin cytoskeleton and can thus sense the mechanical load between them. We previously reported that the Vinculin biosensor protein was recruited to cell junctions of target cells in response to CaLM-induced contractility (Kong et al., 2019). Following CaLM in individual AS cells, we measured changes of VincD1-GFP fluorescence normalized by E-CadmTom (Figure 3). Our measurements revealed an increasing ratiomeric signal at the junction next to but not part of the target cell. Next-next and further distant junctions showed less or no difference in the normalized reporter signal. Ninety seconds after uncaging, we detected a 30% increase at junctions of the target cell, 20% at first junctions and 10% at second neighbor junctions (Figure 3C). In more distant junctions, the reporter signal remained relatively stable and was not different from the reporter signal as distant junctions. In summary, our experiments showed that the optochemically controlled contractility generates a pulling force on the surrounding cells. Consistent with previous reports, we showed that acute pulling forces from the region of the AS next to the epidermis could lead to a displacement of the leading edge.

### Induced Contractions in the Lateral Epidermis Antagonize Dorsal Closure

Having analyzed the AS tissue, we asked whether increased contractility in the lateral epidermis would counteract the pulling by the AS cells. As a preliminary test for a contribution of the epidermal cells to the force balance between AS and epidermis, we increased cell contractility by the expression of constitutively active RhoA (Rho^V14^) (Lee et al., 2000) in four- to five-cell-wide epithelial strips with an *engrailed*-GAL4 driver (Supplementary Figure S2). In the case of a passive contribution and cell elongation as a mere consequence of DC, we would expect no delay of DC. Alternatively, an active contribution would be visible by a delay in DC. Rho^V14^-expressing cells but not in control embryos (Supplementary Figures S2A and B) showed an isotropic cell shape compared to the engrailed negative cells in those embryos (Greig and Bulgakova, 2020). The inhibition of cell elongation indicates that contraction was induced. By the end of embryonic development, we observed the dorsal hole in the cuticles of Rho^V14^-expressing embryos, which may indicate potential DC defects.

**FIGURE 4:**
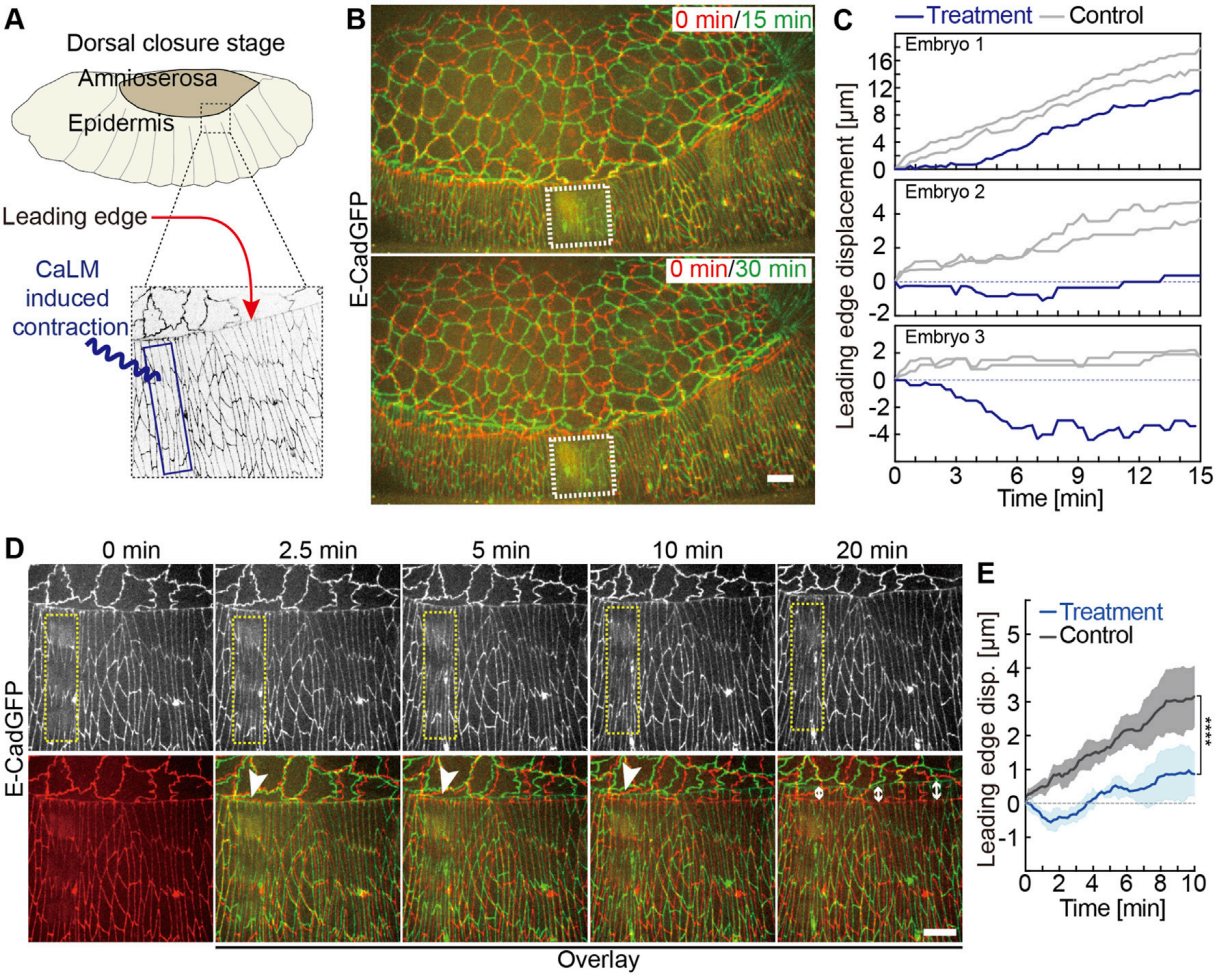
Induced contractility in the lateral epidermis antagonizes dorsal closure. **(A)** Experimental scheme of optochemically (CaLM) induced contraction of lateral epidermal cells locally during dorsal closure in *Drosophila* embryos. The blue box indicates the region of UV illumination, where the cell contraction is triggered optochemically. **(B)** Images from the time-lapse recording of embryos expressing E-CadGFP after optochemically induced contraction in epidermal cells locally. It shows that the optochemically induced lateral epidermal cell contractions temporarily and locally block dorsal closure. Image at 0 min is the first frame just after UV illumination of time-lapse recording. The white dashed boxes indicate the cell contractility region triggered optochemically. **(C)** Quantification of the leading-edge displacement dorsally over 10 min after UV-laser illumination from three individual embryos. The leading edge far away from the optochemically induced contracting epidermal cells in the same embryo was used as control. **(D)** Images from a high-magnification time-lapse recording of an embryo expressing E-CadGFP after optochemically induced contraction in epidermal cells locally. The yellow dashed boxes in the top panels indicate the cell contractility region triggered optochemically. Bottom panels show the images (green) from the top panels overlay with the image at 0 min (red). The white arrowheads indicate that the leading-edge movements are blocked temporarily nearby the optochemically induced contracting cells. The white arrow lines indicate the leading-edge displacements after 20 min, while the lateral epidermal cell contractility was reversed. **(E)** Quantification of the leading-edge displacement dorsally over 10 min after UV-laser illumination. The leading edge far away from the optochemically induced contracting epidermal cells in the same embryo was used as control (*N* = 6 from three embryos, two independent measurements from each embryo). A two-way ANOVA estimates the *p* value. Curves represent the mean (bold line) with SEM (ribbon band). Scale bars are 10 μm.

To better test the hypothesis that lateral epidermal cells actively contribute to DC, we turned to an acute interference instead of the chronic expression of activated RhoA. We applied CaLM to lateral epidermal cells in three rows of cells including the leading-edge cells and avoided the AS-epidermal interface. As a readout, we measured the movement of the leading edge (Figure 4A). We first induced contractions in a region 6–8 cells wide (region of interesting, white dashed boxes in Figure 4B), avoiding the actin cable (Figure 4B). Strikingly, we observed that the leading edge of the target region failed to move dorsally, whereas the distant regions moved as normal (Figures 4B,C). We measured the leading-edge displacements nearby and CaLM application region in three individual embryos after CaLM application. The leading-edge displacements far away from the CaLM application region in the same embryo were measured as controls. The leading-edge movements were blocked, and in some cases, it even moved toward the ventral, and on the contrary, the leading edge in the same embryos far away from the CaLM application region remained to move dorsally (Figure 4C). Scanning the complete region of interest with the UV laser, we bleached, unfortunately, the GFP signal labeling the cell outlines. To follow the shapes of epidermal cells, we applied CaLM with higher-magnification objectives but a smaller field of view. In this way, we spot-illuminated the target cells one by one (Figure 4D). As a consequence of the induced contractility, cell elongation was explicitly inhibited in the target cells (Figure 4D, Supplementary Video S4). The leading edge close to the target cells moved even slightly ventrally for 2.5 min and was blocked there for a couple of minutes and finally started to move dorsally again (white arrowheads in Figures 4D,E). In contrast with control (leading edge in the same embryos far away from the CaLM application region), the leading-edge movements were significantly blocked and even to the opposite direction for 2.5 min due to CaLM-induced contractions in the lateral epidermal cells (Figure 4E). Our data show that optochemically induced contractions in the lateral epidermal cells temporarily and locally antagonized DC. Together with the genetic Rho^V14^ expression data (Supplementary Figure S2), our results suggest that DC requires elongation in epidermal cells.

### Changes in Tissue and Junctional Tension in the Lateral Epidermis During Morphogenesis

**FIGURE 5:**
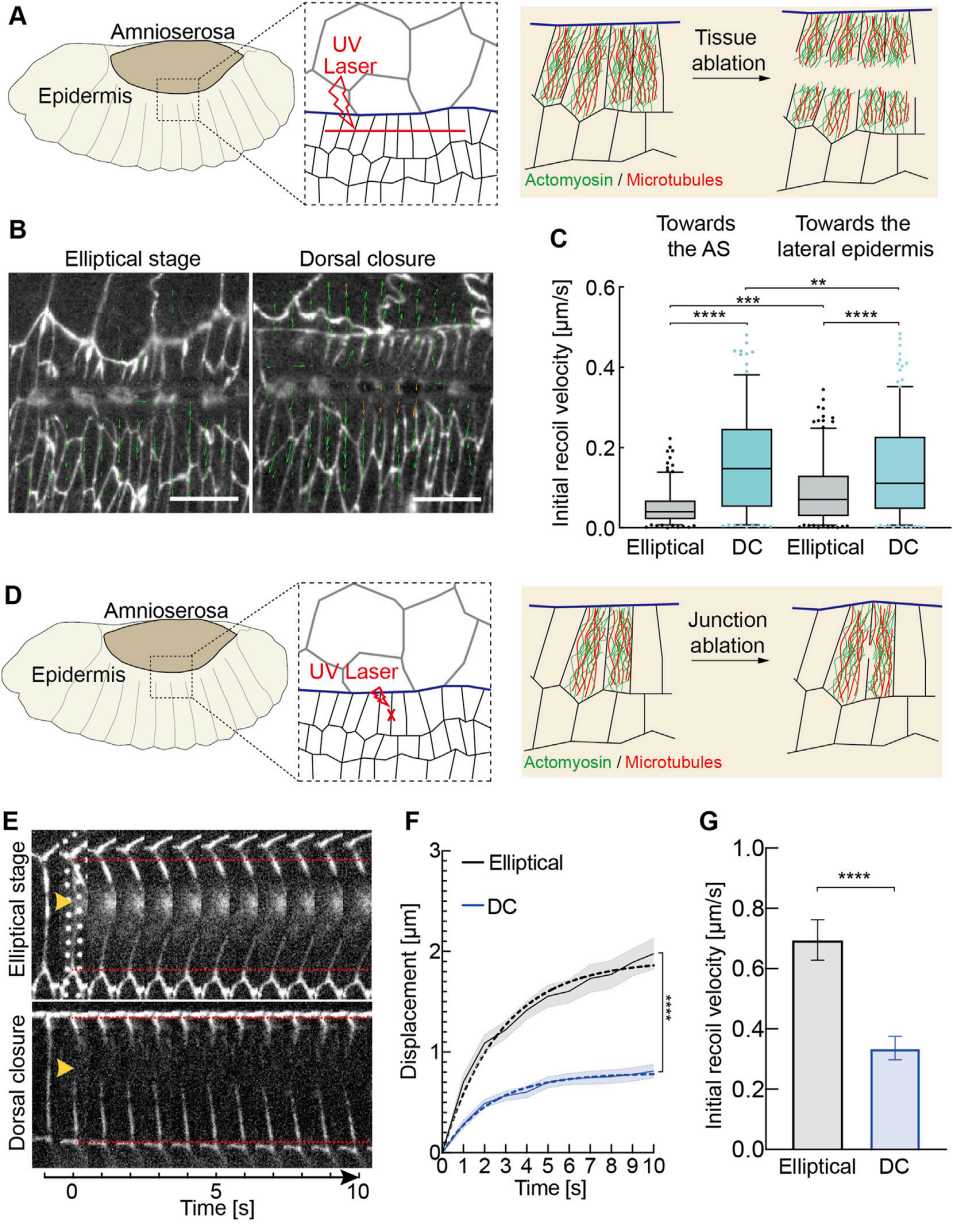
Changes in tissue and junctional tension during morphogenesis. **(A–C)** Developmental up-regulation of tissue tension in the lateral epidermis **(A)** Experimental scheme of tissue laser ablation in the lateral epidermis. A 30 μm linear ablation (red dashed line) was performed along the axis in the lateral epidermis in the region shown in the black dashed box. **(B)** Images from the time-lapse recording after the laser ablation. E-CadGFP outlines cells. The green arrow lines indicate the velocity vectors performed by particle image velocimetry (PIV) analysis. The length of vectors represents the magnitude of the recoil velocity. **(C)** Quantification of the recoil velocity from vector horizontally (axis orientation) and vertically (lateral orientation). Boxplot shows the initial recoil velocities after tissue ablation. Boxes, second and third quartiles; whiskers, 95% confidence interval; horizontal line, mean. Dots are the data outside of confidence interval. Three independent experiments from three embryos in each stage, *N* = 192 particles from each stage toward the AS, and *N* = 213 particles from each stage toward the lateral epidermis. Two-way ANOVA estimates the *p* values between the particles toward the same directions (the AS or the lateral epidermis) from different stages. The *Mann–Whitney* test estimates the *p* values between particles toward the AS and the lateral epidermis from the same stages. **p < 0.01, ***p < 0.001, and ****p < 0.0001. **(D–G)** Developmental down-regulation of initial recoil at individual junctions in the lateral epidermis. **(D)** Experimental scheme of junctional laser ablation in the lateral epidermis. A signal junction in the dorsal-most epidermal (DME) cells along the lateral direction ablated is shown in red. **(E)** Kymographs of representative junction cuts shown in panel **(D)**. **(F)** Vertex displacement versus time plot for junction cuts. Black and blue curves represent the mean with SEM (error bars) of the displacement of junction cuts from the embryos at the elliptical stage and dorsal closure. Black and blue dashed curves represent the one-phase association fitting of experimental data. *N* = 18 junctions from 15 embryos at the elliptical stage and *N* = 15 junctions from 15 embryos at the DC stage, two-way ANOVA, Sidak’s multiple comparison test, ***p < 0.001. **(G)** Mean with the SEM of initial recoil velocities from the one-phase association fitting in panel **(F)**. Unpaired two-sided *t*-test estimates the *p* value, ***p < 0.001.

We employed microsurgical tissue cuts to assay the mechanical tension in the lateral epidermis. Ablation studies suggest that the lateral epidermis is under tension during DC (Kiehart et al., 2000). However, it remains unclear whether the transition in tissue tension occurs in the lateral epidermis. Tension is determined by the ratio of velocity and phenomenological friction. As friction is difficult to measure, we relied on the initial recoil velocity after the cut as a direct measure for tension. We first performed a tissue scale cut parallel to the leading edge in the lateral epidermis (Figure 5A). With particle image velocimetry (PIV), we calculated the initial recoil velocities in both directions toward and away from the AS (Figures 5B,C, Supplementary Video S5). Our measurements revealed larger initial recoil velocities at the DC stage than at the elliptical stage, which was consistent with cell elongation during DC. Our data revealed the developmental upregulation of tissue tension in the lateral epidermis.

We compared the initial recoil velocities after ablation in different directions, toward the AS and lateral epidermis, in each stage. The initial recoil velocities toward the AS are greater than toward the lateral epidermis during the DC stage (Figure 5C). Our data agreed with the previous studies that AS contractions generate pulling forces to the lateral epidermis during DC (Figure 1A) (Solon et al., 2009; Saias et al., 2015). Interestingly, the initial recoil velocities away from the AS were significantly greater than toward the AS during the elliptical stage (Figure 5C). It did not agree with the previous biophysical model in which the tissue tension in AS and lateral epidermis is balanced with each other initially, and a temporal increase in AS surface tension could drive closure (Saias et al., 2015). During the elliptical stage, the pulling force of AS is balanced by the later epidermis since the interface does not move. The force balance changes between the lateral epidermis and AS at the transition from the elliptical stage to DC. The movement during DC indicates that changes in the cell biology of AS lead to an imbalance with the AS taking over or the lateral epidermis giving in (Figure 1A). Our data suggest that the tissue tension in AS and lateral epidermis is not balanced with each other at the elliptical stage.

Next, we measured the tension on the cellular scale. We cut individual junctions of epidermal cells at the leading edge prior to and during DC (Figures 5D,E). Surprisingly, the initial recoil velocities significantly decreased by almost 50% from the elliptical stage to DC, in contrast, to expect given the increased tension on the tissue scale and elongated cells (Figures 5E–G). Our data show that the cellular material properties vary in the lateral epidermis during morphogenesis. The opposite outcome between the tissue and individual junction levels may be due to the experimental procedure. The laser is focused on the cell border for junction cuts, including the cortex with cortical actomyosin (Figure 5D). In contrast, the complete cell, including the cytoplasm and the cytoskeleton, is ablated for tissue cut (Figure 5A).

### Developmental Upregulation of Non-Muscle Myosin II in Lateral Epidermal Cells

**FIGURE 6:**
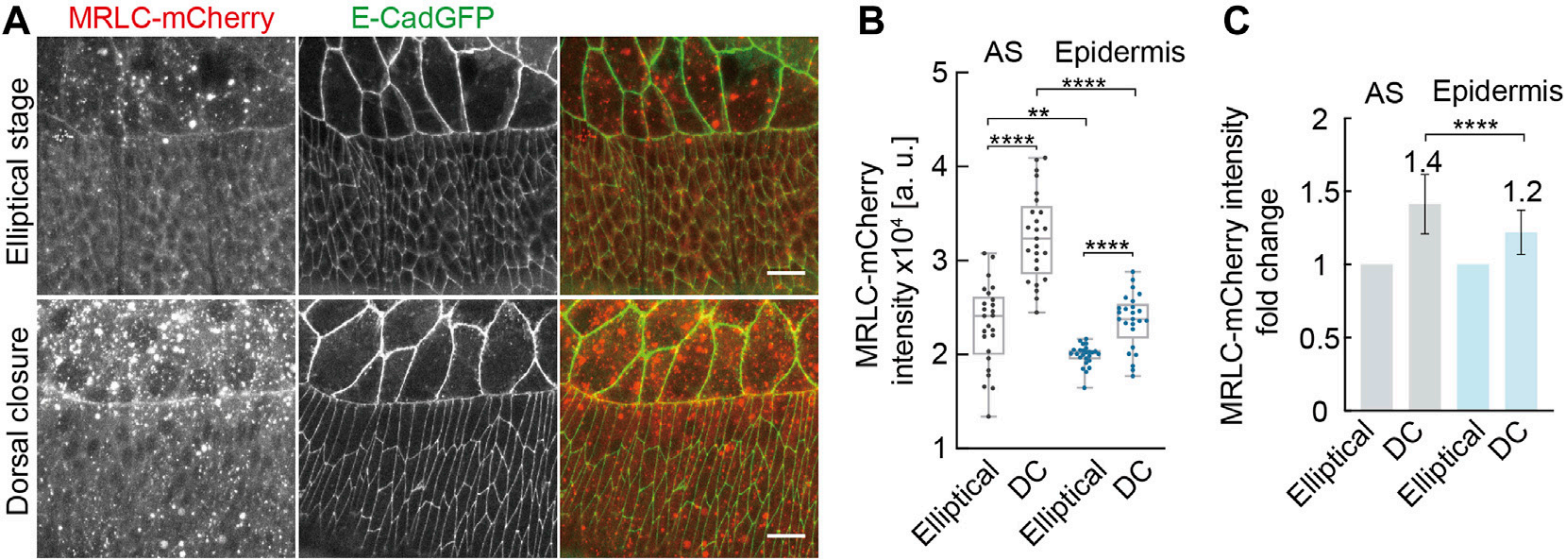
Developmental up-regulation of non-muscle myosin II both in AS and in lateral epidermal cells during morphogenesis. **(A)** Maximum Z-projections of spaghetti squash (the regulatory light chain of the non-muscle myosin II in *Drosophila*) visualized by MRLC-mCherry at the elliptical stage and during dorsal closure. E-CadGFP labels the cell outline. **(B)** Quantification of MRLC-mCherry intensities in AS and lateral epidermal cells at the elliptical stage (Elliptical) and during dorsal closure (DC). For details, see Methods. Boxes, second and third quartiles; whiskers, the min to the max; horizontal line, median; dots, individual data. N = 25 from five embryos, five independent measurements from each embryo from each tissue in each stage. **(C)** Quantification of MRLC-mCherry intensities fold change in AS and lateral epidermal cells. MRLC-mCherry intensities from the elliptical stage embryos were normalized to 1. Two-way ANOVA estimates the *p* values, **p < 0.01, ****p < 0.0001. Scale bars are 10 μm.

We searched for any developmental changes in the cytoskeleton between the elliptical and DC stages. Actomyosin was our first candidate as it is the main regulator for elliptical cell contraction and shape (Koenderink and Paluch, 2018). We examined the levels of active non-muscle myosin II by the fluorescence intensity of an mCherry-tagged spaghetti squash (sqh), the regulatory light chain of the non-muscle myosin II (MRLC) in *Drosophila*, during elliptical and DC stages (Figure 6). We detected increased MRLC-mCherry intensities in AS cells in the DC stage compared to those in the elliptical stage. We also detected a corresponding increase of MRLC-mCherry in the lateral epidermal cells (Figure 6). The averaged, MRLC-mCherry intensities were slightly higher in the AS, and the relative upregulation of ≈1.4-fold in AS and 1.2-fold in the epidermis was significantly different (Figure 6C). Using a phospho-specific myosin antibody (P-Myo) (Losick and Duhaime, 2021), we confirmed the developmental upregulation of active myosin both in AS and in the lateral epidermal cells (Supplementary Figure S3). P-Myo staining was comparable in AS and the lateral epidermal cells at the elliptical stage (Supplementary Figure S3B). Notably, P-Myo staining in the lateral epidermal cells was more intense than in AS cells from DC stage embryos (Supplementary Figure S3B). The average mean intensities of P-Myo staining were upregulated 1.2-fold in AS and 1.7-fold in the lateral epidermis from the elliptical to the DC stage (Supplementary Figure S3C). Consistent with our tissue ablation experiments, our data exclude that the epidermal cell shape change is a simple relaxing process. Furthermore, the opposite results from junctional ablation suggest the transition of other factors from the elliptical stage to AS.

### Developmental Upregulation of Microtubules in Lateral Epidermal Cells

**FIGURE 7:**
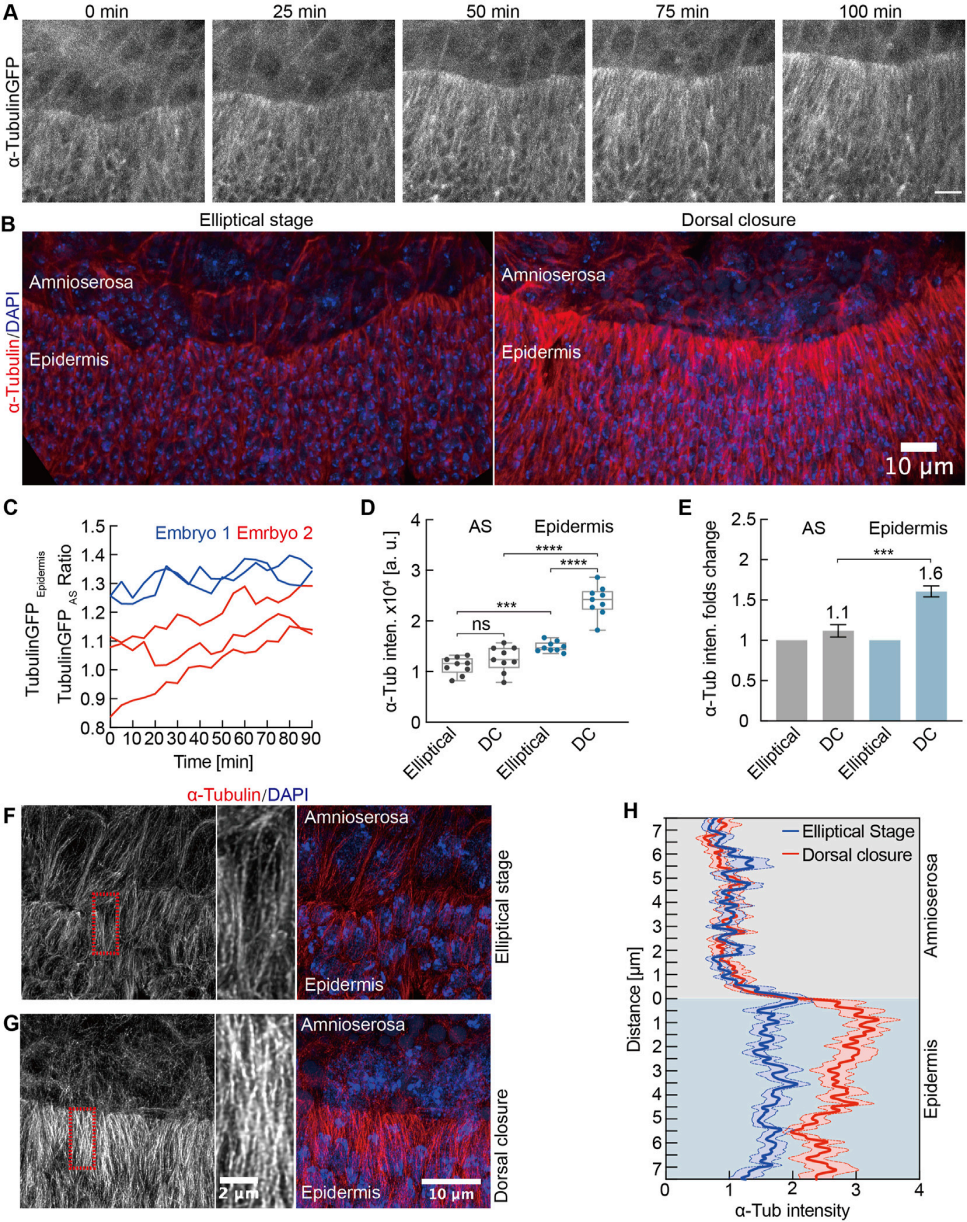
Developmental upregulation of microtubules in lateral epidermal cells. **(A)** Maximum intensity projections from a time-lapse recording of an embryo expressing alpha-Tubulin-GFP undergoing dorsal closure; 0 min, the first frame of the recording. Scale bar, 10 μm. **(B)** Maximum intensity projections of alpha-Tubulin staining during the elliptical stage and dorsal closure. DAPI stained DNA. **(C)** Time course of the alpha-Tubulin-GFP intensities as the ratio between the signal in lateral epidermis and AS. Each curve represents an independent measurement. **(D)** Quantification of alpha-Tubulin intensities in AS and lateral epidermal cells during the elliptical stage and dorsal closure (DC). Boxes, second and third quartiles; whiskers, the min to the max; horizontal line, median; dots, individual data. N = 9 from three embryos, three independent measurements from each embryo from each tissue and stage. **(E)** Quantification of alpha-Tubulin intensities in AS and lateral epidermal cells normalized to the elliptical stage. Two-way ANOVA estimates the *p* values, ***p < 0.001, ****p < 0.0001, ns, nonsignificant. **(F,G)** High-resolution images of the microtubules. Insets in high magnification as indicated by dashed boxes (red). **(H)** Line profiles of alpha-Tubulin staining over the tissue interphase. Mean (bold line) with SEM (ribbon band) of 12 profiles from three embryos at each stage, four independent profiles from each embryo at each stage.

The microtubule cytoskeleton is essential for regulating the cell shape (Dogterom and Koenderink, 2019) and for the fusion of the left and right sheets of the lateral epidermis in the late DC (Takács et al., 2017). We detected a specific upregulation of microtubules in the lateral epidermis by time-lapse imaging and by staining of fixed embryos (Figures 7A,B, Supplementary Video S6). We quantified the intensities of alpha-Tubulin-GFP during the course of DC in both epidermis and AS, revealing an increasing ratio between the epidermis and AS signals over the course of more than an hour (Figure 7C). We measured a corresponding increased staining in the epidermis in fixed embryos (Figures 7D,E). Whereas alpha-Tubulin staining in the AS was similar in the elliptical to the DC stage (factor of 1.1), we detected a significant 1.6-fold increase in the epidermis (Figures 7B,D,E).

Next, we employed a high-resolution imaging with an AiryScan detector to obtain an insight into the organization of microtubules (Figures 7F,G). Whereas microtubules were apparently oriented in the lateral direction during the elliptical stage in both epidermis and AS, the pattern striking changed in AS. During DC, the microtubules appeared isotropic as no preferred orientation was obvious. In contrast, the lateral orientation remained unchanged in the epidermal cells (Figures 7F,G, Supplementary Videos S7 and S8). With these high-resolution images, we quantified staining intensity at the tissue interphase by line profiles. We detected a sharp change of intensity at the tissue interphase during DC but only a slight difference during the elliptical stage (Figure 7H), consistent with the previous quantification.

In summary, we detected a developmental upregulation in microtubules specific for the epidermis. Large-volume electron tomography previously revealed that microtubules orient the protrusions of epidermal cells (Eltsov et al., 2015). The direct interaction of microtubules with F-actin bundles facilitates an initial contact during zippering between epidermal cells from the left and right tissue sheets. Expanding this previous finding, our data suggest a role of microtubules already during the early DC.

## Discussion

The interaction of two or multiple tissues is central to many morphogenetic processes. During ventral furrow formation, for example, contractility of the mesodermal cells induces cell shape changes leading to invagination (Martin et al., 2009; He et al., 2014). The mesoderm pulls on the neighboring ectodermal cells, which respond by specific cell shape changes (Rauzi et al., 2015; Bhide et al., 2021). Correspondingly, the contractile AS pulls on the adjacent epidermis during DC and induces cell elongation. Tissues may communicate by chemical signals and mechanics, at least when epithelia are involved (Heisenberg and Bellaïche, 2013; Collinet and Lecuit, 2021; Roy Choudhury et al., 2021). In most cases, the interactions between the different tissues, the corresponding cell types, and their role in morphogenesis have been little investigated.

In this study, we artificially induced contractions by genetically upregulating RhoA in epithelial strips. The progression of DC was antagonized at the sites of increased contractility in these embryos. Although cell shape and the actin cable were impacted in these embryos, a previous study concluded that DC was disrupted due to the affected actin cable (Jacinto et al., 2002a), albeit a contribution by the different cell shapes was not ruled out. More informative and conclusive were acutely induced cell contractions by CaLM (Ca^2+^-mediated light activation of non-muscle myosin II). Contracting epidermal cells transiently reversed cell elongation and DC, indicating a balance of forces at the tissue interface. An increased contractility on any of the two sides will shift the balance in that direction.

The squamous AS cells prominently display area oscillations. Based on the pattern of AS cell contraction, only a fraction of AS cells contribute to the tissue contractility at every time point during DC (Solon et al., 2009). A prediction is that additional contractility in the AS will increase the pull on the lateral epidermis. We demonstrated by CaLM-induced AS contraction that additional contractility speeds up the movement of the tissue interface. The ratchet model for DC proposes pulsed apical AS cell surface contractions transiently pulled on the neighboring epidermis (Solon et al., 2009). We would expect a corresponding pulsatile behavior in the epidermis based on this model. Indeed, we detected a pulsatile elongation of epidermal cells. The epidermal cells might be passively stretched by the pulling forces by the AS contraction. The non-muscle myosin II-dependent contractions within the AS are required for DC (Pasakarnis et al., 2016). A contribution by the epidermal cell elongation has remained unclear, however, awaiting a more systematic and detailed analysis. Interestingly, the elongation of epidermal cells is still observed in *Zasp52* mutant embryos, which lack any actin cable but undergo an apparently normal DC (Ducuing and Vincent, 2016). This report suggested that the actin cable is not the dominant factor for lateral epidermal cell elongation.

We detected increased non-muscle myosin II levels in the lateral epidermis during DC compared to the preceding elliptical stage embryos. Correspondingly, we detected increasing tissue tension from the elliptical stage to DC. Our data reveal a developmental upregulation of tissue tension in the lateral epidermis, which counteracts the pull from the AS. Our data do not support a passive model in that a “relaxed” lateral epidermis simply follows the pull by AS contracting forces. Our experiments provide further support for an active balance model with induced contractility. Additional contractility in the epidermis antagonizes DC. Both up- and down-regulation of the Rho signaling in epithelial strips affect DC (Jacinto et al., 2002a). DC was completed in the embryos that restricted the non-muscle myosin II depletion in the lateral epidermis but delayed (Pasakarnis et al., 2016). Developmental upregulation of tissue tension in the lateral epidermis might be helpful for the robustness of DC.

In contrast to tissue-wide tension, we detected a decreased tension at individual junctions from the elliptical stage to DC. The divergence of tension from tissue to junction suggests that structures are involved in bearing the pull by the AS, which are not ablated by the junction cuts, such as cytoplasmic components and large parts of the microtubule cytoskeleton. We assumed that the microtubule cytoskeleton may be an active factor in the elongation of the lateral epidermal cells. We detected a striking rearrangement of the microtubule cytoskeleton in the lateral epidermal cells. The decreasing junction tension in DC may be due to the microtubules, which are affected in tissue cuts but less so in precise cuts of individual junctions (Lv et al., 2020). The microtubule cytoskeleton plays a vital role in regulating cell shape (Dogterom and Koenderink, 2019). Microtubule organization changes along with the shape in epithelial cells during *Drosophila* embryogenesis (Gomez et al., 2016). Our results suggest a hypothesis that the lateral epidermal cells elongate initially due to the AS contractility forces, and microtubule alignment responds to cell elongation and stabilize the elongated cells. The detailed functions of the microtubule cytoskeleton for DC and the lateral epidermal cell need to be investigated further in the future.

The material properties of cells are critical components for cell dynamics (Barriga and Mayor, 2019; Corominas-Murtra and Petridou, 2021). Increasing evidence suggested that tissue and cells undergo material property change during morphogenesis (Petridou and Heisenberg, 2019). The viscoelasticity of cells affects several single-cell functions such as shaping, division, and motility, which are largely determined by the physical properties of the underlying cytoskeletal network (Pegoraro et al., 2017). The epidermal cells undergo cell elongation and changes of viscoelastic deformation during morphogenesis in the *Drosophila* wing (Iyer et al., 2019). Our ablation experiments demonstrate material property changes in the lateral epidermis during development.

## Materials and Methods

### *Drosophila* Genetics

The following transgenes were used: E-Cadherin-GFP (Huang et al., 2009), E-Cadherin-mTomato (Huang et al., 2009), MRLC-mCherry (Martin et al., 2009), and UAS-Vinculin D1-GFP (Kong et al., 2019). The following transgenes are from the Bloomington *Drosophila* Stock Centre (Whitworth, 2019), and a Flybase Symbol (Thurmond et al., 2019) for each transgene is provided here*. engrailed*-GAL4 (Bloomington number 30564, Flybase Symbol, Dmel\P{en2.4-GAL4}e16E), AS-Gal4 (Bloomington number 3734, Flybase Symbol, Dmel\P{GawB}c381), alpha-Tubulin-GFP (Bloomington number 7253, Flybase Symbol, Dmel\P{GAL4:VP16-nos.UTR}CG6325^MVD1^), and UAS- Rho^V14^ (Bloomington number 7330, Flybase Symbol, Dmel\P{UAS-Rho1.V14}5.1) were used.

### Larval Cuticle Preparations

First instar larvae were dechorionated with bleach, washed with water, mounted in a 1:1 (v/v) mixture of Hoyer’s medium (Hoyer, 1882) and lactic acid, and incubated at 65°C overnight. Photographs were taken with the darkfield illumination.

### Histology and Microscopy

Embryos were fixed, stained, and mounted as previously described (Zhang et al., 2014). Embryos were fixed by 37% formaldehyde for alpha-Tubulin staining. Antibodies against the following antigens were used: engrailed (mouse anti-Engrailed, 4.7 μg/mL, Hybridoma center 4D9), DCAD2 (rat anti-E-Cadherin, 7 μg/mL, Hybridoma center) (Oda et al., 1994), P-Myo (Rabbit anti-Phospho-myosin, 1:200, Cell Signaling), and alpha-Tubulin (mouse anti-alpha-Tubulin, 8 μg/L, Sigma B512). Secondary antibodies were labeled with Alexa dyes (Invitrogen, 5 μg/mL). GFP was stained by a nanobody labeled with ATTO 488 dye (GFP-Booster, chromotek). DNA was stained by DAPI (Sigma). Specimens were mounted in Aqua polymount (Polysciences).

Fluorescent images from fixed embryos were acquired on a laser scanning confocal microscopy (Carl Zeiss, ZEISS LSM 980 with Airyscan 2). Images in Figure 6 and Supplementary Figure S3 were acquired with a ×63 oil objective (Carl Zeiss, ×63/oil, NA1.4) with 0.5 μm intervals for Z-stacks. Images in Figure 7B and S2 were acquired with a ×40 water objective (Carl Zeiss, ×40/W, NA1.2) with 0.5 μm breaks for Z-stacks. For super-resolution imaging (Figures 7F,G and Supplementary Videos S7, S8), images were acquired with a ×63 oil objective (Carl Zeiss, ×63/oil, NA1.4, Airyscan model) with 0.17 μm interval for Z-stacks and processed by a ZEISS Airyscan Joint Deconvolution (Carl Zeiss). Image stacks were processed with “max intensity” in ImageJ/Fiji (Schindelin et al., 2012). 3D reconstruction was carried out by Imaris9 (Supplementary Videos S7, S8).

### Live Imaging

Embryos were prepared as described previously (Kong et al., 2019). Briefly, the staged embryos were collected and dechorionated with 50% hypochlorite bleach for 90 s, aligned on an agar block, and attached on the coverslips by homemade glue covered with halocarbon oil. Cross-sectional images were recorded from the dorsal side for E-CadGFP (488 nm excitation) on a spinning disc microscope with a 40x or ×100 oil objective (Carl Zeiss, ×40/oil, NA1.2, 100x/oil, NA1.4) with an emCCD camera (Carl Zeiss, AxioCam MRm). The apical plane of the embryo was acquired with axial sections of each 0.5 µm and a frame rate of 0.2/min or 1/min. Images in Figure 6A were acquired on a laser scanning confocal microscopy (Carl Zeiss, ZEISS LSM 980 with Airyscan 2) with a ×63 oil objective (Carl Zeiss, ×63/oil, NA1.4), and the apical plane of the embryo was acquired with axial sections of each 0.5 µm. Images in Figure 7A were acquired on a laser scanning confocal microscopy (Carl Zeiss, ZEISS LSM 980 with Airyscan 2) with a ×63 oil objective (Carl Zeiss, ×63/oil, NA1.4), and the apical plane of the embryo was acquired with axial sections of each 1 µm and a frame rate of 0.2/min.

### Optochemically Induced Contraction (CaLM: Ca^2+^-Mediated Light Activation of Non-Muscle Myosin II)

Embryos were prepared as described previously (Kong et al., 2019). Briefly, embryos were collected and dechorionated with 50% hypochlorite bleach for 90 s, dried in a desiccation chamber for ∼10 min, covered with halocarbon oil, and injected dorsally into the vitelline space in the dark at room temperature (∼22°C). After injection, the embryos were incubated at room temperature in the dark for about 10 min before uncaging. Nitrophenol-EGTA, AM (NP-EGTA ester, Invitrogen) was prepared in a 1x injection solution (180 mM NaCl, 10 mM HEPES [pH 7.2], 5 mM KCl, 1 mM MgCl_2_); 1 mM NP-EGTA, AM was injected for Ca^2+^ uncaging in AS cells (Figures 2, 3, Supplementary Figure S2, and Supplementary Video S3), and 2 mM NP-EGTA, AM was injected for Ca^2+^ uncaging in epidermal cells (Figure 4 and Supplementary Video S4).

A 375 nm diode laser (DPSL-375, up to 200 mW, Rapp OptoElectronic) was employed for Ca^2+^ uncaging. The laser was mounted on the epiport of a spinning disc microscope (Zeiss). The laser was applied on the “REO-SysCon-Zen” platform (Rapp OptoElectronic), while the images were recorded on a spinning disc microscope with a ×100 oil objective (Carl Zeiss, ×100/oil, NA1.4) with a CCD camera. An emCCD camera (Photometrics, Evolve 512) was used in Supplementary Figure S3.

For cell dynamics, cross-sectional images were recorded in the GFP channel (488 nm excitation) with a frame rate of 0.2/s (one frame every 5 s), immediately following uncaging. Flies carried the UASt-VincD1GFP; E-cad-mTomato transgenes were crossed with the AS-Gal4 driver flies. Embryos from this cross expressing VincD1GFP in AS cells were employed for uncaging. Cross-sectional images were recorded from the dorsal side in the GFP channel (488 nm excitation) with a CCD camera at a frame rate of 1/s, immediately following uncaging. The recording was switched to the emCCD camera with a 488 nm excitation for VincD1GFP and a 561 nm excitation for E-Cad-mTomato. The GFP and mTomato channels were recorded simultaneously at a frame rate of 0.1/s. The apical plane of the embryo was acquired with four axial sections of each 0.5 µm.

### Laser Ablation

Stage 13 or 14 embryos expressing E-Cad-GFP were prepared as live imaging. Cross-section images were recorded in the GFP channel with a frame rate of 1/s on a spinning disc microscope (Zeiss, 100x/oil, NA1.4) with a CCD camera. A 355 nm pulsed laser (DPSL355/14, 355 nm, 70 µJ/pulse, Rapp OptoElectronic) was employed for ablation and manipulated on the “REO-SysCon-Zen” platform (Rapp OptoElectronic). The laser was mounted on the epiport of a spinning disc microscope.

Tissue laser ablation was applied with a 30 μm line along the axis in the lateral epidermis with 5% of laser power during the recording mode. Particle image velocimetry (PIV) of E-CadGFP images after tissue laser ablation (Figures 5A–C and Supplementary Video S5) analysis was performed using square interrogation windows of side 64 pixels by the “PIVlab” (Thielicke and Sonntag, 2021) in MATLAB.

Junction ablation was performed with 5% of laser power and a 200 ms (around 40 pulses) exposure time during the recording mode (100x oil, NA 1.4) (Figures 5D–G). The displacement of both ends (L(t)) of ablated junctions was measured manually in Fiji/ImageJ. The displacement (Figure 5F) was fitted as a Kelvin–Voigt fiber model (Fernandez-Gonzalez et al., 2009) to the Eq. 1 on Prism8 (Liang et al., 2016).

### Extraction of Initial Recoil Values

$$\text{ε}\left(\text{t}\right)=\text{L}\left(\text{t}\right)-\text{L}\left(0\right)=\frac{{\text{F}}_{0}}{\text{E}}.\left(1-e^{-\left[\left(\frac{\text{E}}{\text{μ}}\right)\text{*t}\right]}\right)$$ (1)

where

F_0_ is the tensile force present at the junction before ablation, E is the elasticity of the junction, and *μ* is the viscosity coefficient related to the viscous drag of the cell cytoplasm.

Extraction of initial recoil values (Figure 5G) as fitting parameters for the Eq. 2 was introduced.

As fitting parameters for the above equation was introduced

$$initial\;recoil=\frac{d\text{ε}\left(0\right)}{dt}=\frac{F_{0}}{\text{μ}}$$ (2)

### Image Analysis

The image stacks were processed with “max intensity” in ImageJ/Fiji for the leading-edge movement and cell elongation analyses (Figures 1, 2, 4 and Supplementary Figure S1). The leading edge was measured manually in ImageJ/Fiji every frame. Including leading-edge cells, three rows of the lateral epidermal cells from dorsal to ventral in each embryo were analyzed. Five cells from each row were selected randomly for measurements. The cell length was measured along the dorsal–ventral direction manually in ImageJ/Fiji every frame. All the lengths were normalized by *L*_*0*_ (cell length at 0 min, the onset of DC).

To analyze cell dynamics after CaLM (Figures 2, 3 and Supplementary Figure S2), the projected cross-sectional images were filtered with a Gaussian filter and then segmented and tracked with a “Tissue Analyzer” (Aigouy et al., 2016) in ImageJ/Fiji. Cell area measurements were carried out with ImageJ/Fiji. The value of cell area from A_0_ (the first frame after UV illumination) was set to 1, and all the cell areas were normalized by A_0_.

To analyze the VinculinD1-GFP to E-Cadherin-mTomato ratio (Figure 3), the image stacks were processed with “max intensity” under ImageJ/Fiji. The ratio was generated by plugin “Ratio plus” in ImageJ/Fiji, followed by the manual measurements of the intensity. The mean integrated density was measured, as shown in Figure 3, and normalized as follows:

$$I=\frac{I_{t}}{I_{0}}$$ (3)

where the *I*_*0*_ is the measurement from the first frame.

To analyze the intensities of MRLC-mCherry (for non-muscle myosin II in Figure 6), the image stacks were processed with “max intensity” in ImageJ/Fiji. Images from five embryos at each stage were performed. The mean gray value for each measurement was performed in a 12.5 × 12.5 µm box from the AS or the lateral epidermis in ImageJ/Fiji. Five independent measurements were performed in each stage embryo and each tissue. The regions for measurements were selected randomly, while the AS and lateral epidermis interface were avoided.

To analyze the intensity of alpha-Tubulin-GFP (Figures 7A,C), the image stacks were processed with “max intensity” in ImageJ/Fiji. The mean gray value for each measurement was performed in a 12.5 × 12.5 µm box from the AS or the lateral epidermis. The regions for measurements were selected randomly and consistent in each frame, while the AS and lateral epidermis interface were avoided.

Mixtures of embryo were stained in the same tubes, imaged with the same microscopic settings, and images were processed and quantified in parallel (Figures 7 and Supplementary Figure S3). For alpha-Tubulin staining in Figures 7B,D,E, the mean gray value for each measurement was measured in a 15 µm × 15 µm box in three embryos, whose position was selected randomly while avoiding the tissue interphase. Three independent measurements were conducted for each stage and tissue. In the case of the phosphorylated regulatory light chain of the myosin II (P-Myo) (Supplementary Figure S3), we measured the mean gray value in a 10 μm × 10 µm box in five embryos for each stage embryo and tissue, again avoiding the tissue interphase.

The ratio of fluorescence intensities in Figures 6C, 7E, and Supplementary Figure S3C was calculated by dividing the mean from the elliptical stage with each independent measurement from the DC stage. Numbers were normalized to the mean intensity from the elliptical stage separately for both tissues.

For line profiles across the tissue interphase (Figure 7H), a line with a width of 10 pixels was place over the tissue interphase. Twelve line profiles from three embryos and aligned to the interphase were averaged for each of the two stages.

### Statistics

Statistical analysis was performed with Prism8. Two-way ANOVA and Sidak’s multiple comparison test estimate the *p* values in Figures 2D, 4E, 5F. Unpaired two-sided *t*-test estimates the *p* values in Figures 3, 5G. Two-way ANOVA estimates the *p* values in Figures 6, 7, and Supplementary Figure S3. In Figure 5C, two-way ANOVA estimates the *p* values between the particles toward the same directions (the AS or the lateral epidermis) from different stages; The *Mann–Whitney* test estimates the *p* values between particles toward the AS and the lateral epidermis from the same stages.

## Data Availability

The raw data supporting the conclusions of this article will be made available by the authors without undue reservation.
