# Supplementary figures and images for: The Lateral Epidermis Actively Counteracts Pulling by the Amnioserosa During Dorsal Closure
Source: Front Cell Dev Biol. 2022 May 16;10:865397. doi: 10.3389/fcell.2022.865397 (PMC9148979; doi:10.3389/fcell.2022.865397)

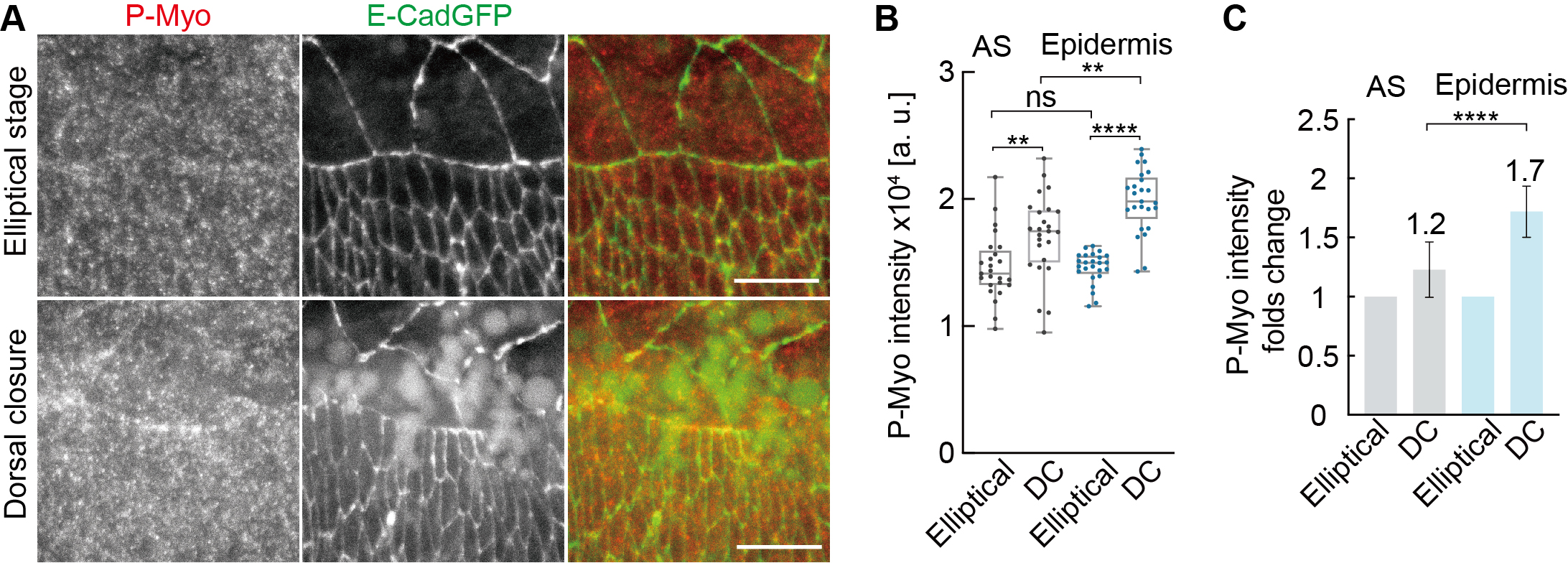

Supplement: Supplementary file 1 [file Image3.JPEG]

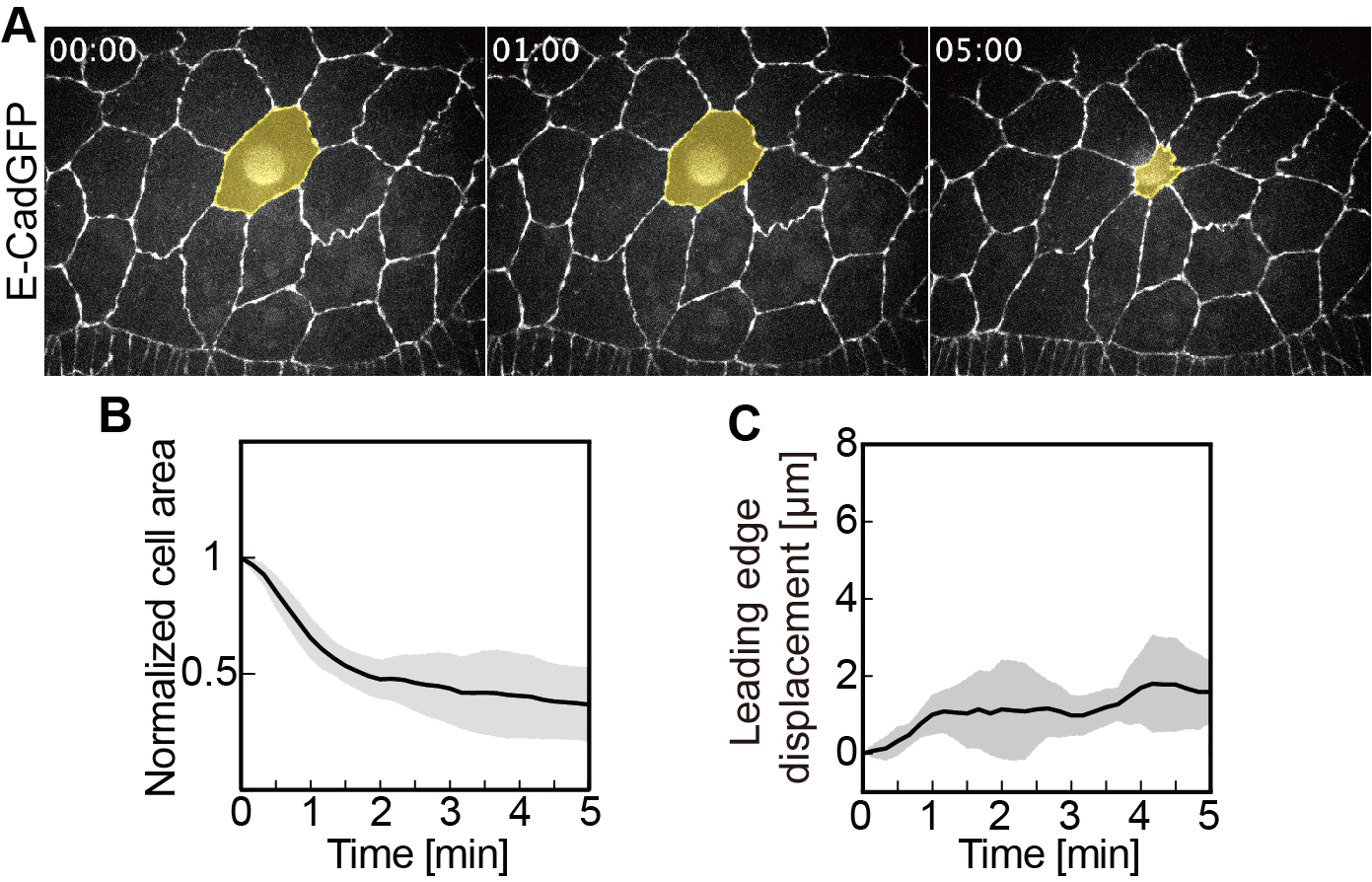

Supplement: Supplementary file 5 [file Image1.JPEG]

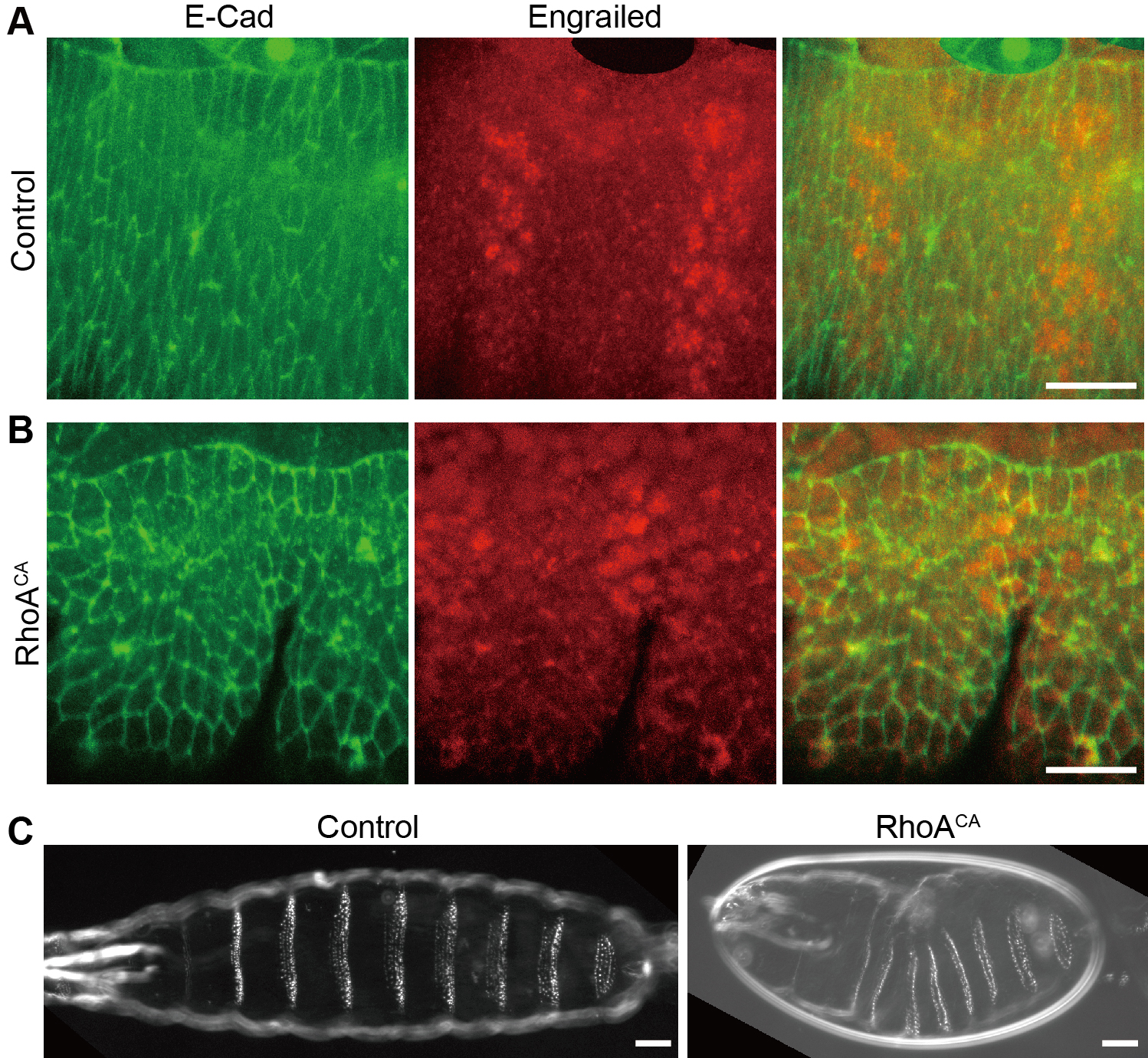

Supplement: Supplementary file 6 [file Image2.JPEG]
